# Recombinant SARS-CoV-2 genomes circulated at low levels over the first year of the pandemic

**DOI:** 10.1093/ve/veab059

**Published:** 2021-07-15

**Authors:** David VanInsberghe, Andrew S Neish, Anice C Lowen, Katia Koelle

**Affiliations:** Department of Biology, Emory University, Atlanta, 1510 Clifton Rd, Atlanta, GA, 30322 USA; Department of Microbiology and Immunology, Emory University School of Medicine, 100 Woodruff Circle, Atlanta, GA, 30322 USA; Department of Pathology, Emory University School of Medicine, 1364 Clifton Rd, Atlanta, GA, 30322 USA; Department of Pathology, Emory University School of Medicine, 1364 Clifton Rd, Atlanta, GA, 30322 USA; Department of Microbiology and Immunology, Emory University School of Medicine, 100 Woodruff Circle, Atlanta, GA, 30322 USA; Emory-UGA Center of Excellence for Influenza Research and Surveillance (CEIRS), Atlanta, GA, USA; Department of Biology, Emory University, Atlanta, 1510 Clifton Rd, Atlanta, GA, 30322 USA; Emory-UGA Center of Excellence for Influenza Research and Surveillance (CEIRS), Atlanta, GA, USA

**Keywords:** SARS-CoV-2, recombination, genome, coronavirus, co-infection

## Abstract

Viral recombination can generate novel genotypes with unique phenotypic characteristics, including transmissibility and virulence. Although the capacity for recombination among betacoronaviruses is well documented, recombination between strains of Severe Acute Respiratory Syndrome Coronavirus 2 (SARS-CoV-2) has not been characterized in detail. Here, we present a lightweight approach for detecting genomes that are potentially recombinant. This approach relies on identifying the mutations that primarily determine SARS-CoV-2 clade structure and then screening genomes for ones that contain multiple mutational markers from distinct clades. Among the over 537,000 genomes queried that were deposited on GISAID.org prior to 16 February 2021, we detected 1,175 potential recombinant sequences. Using a highly conservative criteria to exclude sequences that may have originated through *de novo* mutation, we find that at least 30 per cent (*n* = 358) are likely of recombinant origin. An analysis of deep-sequencing data for these putative recombinants, where available, indicated that the majority are high quality. Additional phylogenetic analysis and the observed co-circulation of predicted parent clades in the geographic regions of exposure further support the feasibility of recombination in this subset of potential recombinants. An analysis of these genomes did not reveal evidence for recombination hotspots in the SARS-CoV-2 genome. While most of the putative recombinant sequences we detected were genetic singletons, a small number of genetically identical or highly similar recombinant sequences were identified in the same geographic region, indicative of locally circulating lineages. Recombinant genomes were also found to have originated from parental lineages with substitutions of concern, including D614G, N501Y, E484K, and L452R. Adjusting for an unequal probability of detecting recombinants derived from different parent clades and for geographic variation in clade abundance, we estimate that at most 0.2–2.5 per cent of circulating viruses in the USA and UK are recombinant. Our identification of a small number of putative recombinants within the first year of SARS-CoV-2 circulation underscores the need to sustain efforts to monitor the emergence of new genotypes generated through recombination.

## 1. Introduction

Severe Acute Respiratory Syndrome Coronavirus 2 (SARS-CoV-2) emerged in December of 2019 in China but has since spread worldwide. Laboratories around the world have been sequencing and rapidly sharing SARS-CoV-2 genomes throughout the pandemic, providing researchers the rare opportunity to study the evolution of SARS-CoV-2 in great detail. As of 16 February 2021, >500,000 complete viral genomes were available on the online repository Global Initiative on Sharing All Influenza Data (GISAID; Elbe and Buckland-Merrett 2017).

In addition to point mutations and insertions/deletions, coronavirus evolution is heavily driven by recombination (Su et al., 2016). Recombination events create chimeric genotypes between two viral strains that infect the same cell. This process occurs when RNA polymerase prematurely stops replicating the first genotype before reassembling and resuming replication with the second genotype as template. The end result is the unlinking of mutations across the genome, creating novel combinations of existing mutations. The clinical and epidemiological relevance of these new combinations is substantial as they have the potential to create genotypes with unique virulence and transmissibility characteristics.

Measurements of the frequency of recombination among coronaviruses in cell culture suggest it is very common (Schaad et al., 1990; Banner and Mc Lai 1991; Gribble et al., 2021), and a recent phylogenetic analysis of seasonal coronaviruses OC43, NL63, and 229E indicates that coronaviruses circulating in humans have undergone extensive recombination (Müller, Kistler, and Bedford 2021). There have further been attempts to detect and measure the magnitude of recombination among naturally circulating SARS-CoV-2 genomes. Based on four single nucleotide polymorphisms (SNPs), an early analysis reported recombinants among the first 85 sequenced SARS-CoV-2 genomes (Yi 2020). Four more recent pre-prints have also identified recombinant SARS-CoV-2 genomes, but used substantially different methods (Korber et al., 2020a; Varabyou et al., 2020; Ignatieva, Hein, and Jenkins 2021; Jackson et al., 2021). Although these analyses have identified recombinant SARS-CoV-2 genomes, four studies have also reported evidence of strong linkage disequilibrium among polymorphic sites, indicating that recombinant SARS-CoV-2 strains are not widespread (Maio et al., 2020; Nie et al., 2020; Richard et al., 2020; Wang et al., 2020).

Here, we add to these existing studies by implementing a lightweight approach based on clade-defining mutational markers to rapidly screen for SARS-CoV-2 genomes that may have originated through the process of recombination. Using this approach, we identify a preliminary set of 1175 potentially recombinant genomes. We analyze the genomes in this set in further detail to parse out the subset that may have originated through *de novo* mutation at these clade-defining sites, leaving 358 genomes that are likely to have originated through the process of recombination. Analysis of deep-sequencing data, where available, indicates that few of these are likely a result of sample contamination or technical sequencing/variant calling error. Given these results, and accounting for limitations in the ability to detect recombination events between genetically similar sequences, we estimate that at most 0.2–2.5 per cent of SARS-CoV-2 infections in the UK and USA are due to recombinant viruses.

## 2. Results

### 2.1 The limited genome-wide diversity among SARS-CoV-2 strains restricts the ability to detect recombinants

Compared to RNA viruses that have been endemically circulating in humans, SARS-CoV-2 in its first year of global circulation harbored only a small amount of genetic variation. As of 16 February 2021, of the approximately 30 thousand sites in the SARS-CoV-2 genome, only 121 positions had nucleotide variants that were present in at least 1 per cent of sequenced genomes, and only 15 positions had variants present in at least 10 per cent of genomes. Consequently, the clade structure of SARS-CoV-2 in the virus’s first year of circulation was overwhelmingly determined by a small number of variant sites. An efficient, lightweight approach to screen for potential recombinant SARS-CoV-2 genomes could therefore rely on detecting unusual combinations of SNPs that were phylogenetically informative during this time.

**Figure 1.**
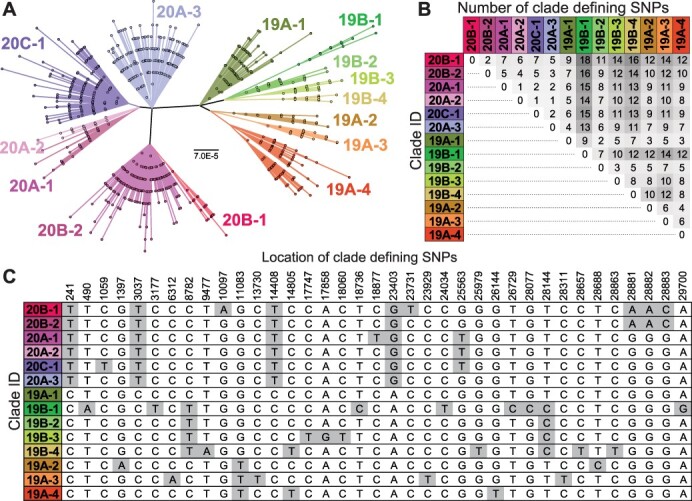
The clade structure of SARS-CoV-2 is shaped predominantly by 37 cdSNPs. (A) An unrooted, maximum likelihood phylogeny based on the General Time Reversible model with invariant sites of 6,536 high-quality unique genome sequences with <1 per cent Ns. The high-quality sequences used spanned collection dates between January 22 2020 and 17 May 2020. Fourteen monophyletic clades with ≥50 per cent bootstrap support were identified and named based on Nextstrain nomenclature (Hadfield et al., 2018). Clade boundaries are in full agreement with Nextstrain clades (e.g. 20B), but some are more finely differentiated for higher resolution in our analyses (e.g. 20B-1). Scale bar is in substitutions per site. (B) Pairwise differences between the cdSNP profiles of all 14 clades. To avoid redundancy, only half of the symmetrical entries are shown. (C) Genomic locations and nucleotide identities of cdSNPs.

To define phylogenetically informative variant sites, we identified SNPs that are strongly associated with major phylogenetic clades within SARS-CoV-2. Multiple nomenclature schemes have been introduced to describe the major lineages that are in circulation; of which the most widely used are the Nextstrain (Nextstrain: Genomic Epidemiology of Novel Coronavirus 2021), GISAID (GISAID 2021), and Pangolin (Rambaut et al., 2020) systems. While Pangolin provides a fine-scaled classification of viral diversity, Nextstrain and GISAID provide broad-scale classification schemes that cluster SARS-CoV-2 genomes into a relatively small number of distinct clades, particularly during the first year of the virus’s circulation. Here, we relied on Nextstrain’s classification scheme to detect genomes collected prior to mid-February 2021 that are potentially recombinant, but increase its resolution to 14 monophyletic clades with ≥50 per cent bootstrap support to expand the number of variant sites that contribute to our detection of potential recombinant genomes (Fig. 1A). We then screened all polymorphic sites in a genome alignment of over 6,000 high-quality genome sequences to identify SNPs that are strongly associated with these 14 clades. In total, we identified 37 clade-defining SNPS (cdSNPs) that reliably distinguish all 14 clades (Fig. 1A–C). Among these positions is the nucleotide substitution of adenine (A) to cytosine (C) at cdSNP site 23,403, which is responsible for the D614G mutation in the spike protein that has been associated with increased transmissibility (Korber et al., 2020b; Plante et al., 2020; Zhou et al., 2021). Of the over half a million complete, human-derived SARS-CoV-2 strains available on GISAID as of 16 February 2021, fewer than 0.5 per cent of genomes differed from the 14 clades’ cdSNP profiles by more than one nucleotide.

Identification of early recombinant genomes is difficult because the clade structure of SARS-CoV-2 in the first year of its circulation was driven by such a limited number of SNPs. The limited numbers of SNPs that distinguish certain clades are also often clustered in short regions of the genome, further restricting our ability to reliably identify potentially recombinant genomes. For instance, clades 20B-2 and 20A-2 are primarily distinguishable based on four cdSNPs (25,563 and 28,881–3), but those four positions span only 3.3 kb of the viral genome. As a result, recombination between strains from clades 20B-2 and 20A-2 throughout the first 80 per cent of the genome would not be detectable. Further, a recombination event that unlinks the nucleotides at Positions 25,563 and 28,881–3 could be parsimoniously explained as a *de novo* T to G mutation at Position 25,563 in a clade 20A-2 genome or a *de novo* G to T mutation at this position in a Clade 20B-2 genome.

Nevertheless, there are many circumstances where detection of recombinants should be feasible. In particular, all major clades are most strongly differentiated from each other based on 11 cdSNPs that are distributed throughout the genome (cdSNP sites 241, 3,037, 8,782, 11,083, 14,408, 23,403, 25,563, 28,144, and 28,881–3). Rearrangement of multiple of these clade-defining markers would be among the strongest indication of recombination between SARS-CoV-2 strains. For instance, the triple mutation GGG to AAC at Positions 28,881–3 is uniquely found in clade 20B and would be a strong marker of recombination between Clade 20B and Clades 19A and 19B when combined with Positions 241, 3,037, 14,408, and 23,403.

### 2.2 Methods that rely on phylogenetically uninformative SNPs are prone to error in detecting recombination

The first report of recombination among SARS-CoV-2 genomes was a correspondence article (Yi 2020), prepared at a time when there were 85 SARS-CoV-2 genomes in GISAID. This article argued that the distribution of four SNPs in those early genomes could be explained by multiple recombination events. With such little phylogenetic information, it is difficult to evaluate the strength of these claims. However, three of the four sites on which they base their argument are, by our analysis, associated with discrete clades and their distribution is readily explained via a clonal pattern of descent. The remaining site (C29095T) is not one of the cdSNPs we identified but is a low-frequency allele that is found in multiple clades (Hadfield et al., 2018) and is thus very likely a homoplasy. Consequently, inference of recombination in this study may have been biased by a low sample number and the use of phylogenetically uninformative SNPs.

**Figure 2.**
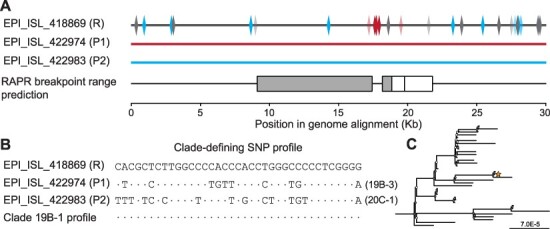
Recombination detection programs designed for low-diversity settings without clade structure have the potential to falsely identify SARS-CoV-2 sequences as recombinants. (A) Three genomes from Washington State, USA, that RAPR identifies as potentially parent and recombinant sequences (*P* = 0.0002). Boxes in the RAPR breakpoint range prediction indicate the ranges where the breakpoint intervals most likely fall; gray boxes indicate the statistically significant range. SNPs in the predicted recombinant sequence (R) that match each parent sequence are shown in red (P1) and blue (P2), while SNPs that do not match either parent sequence are shown in gray. The 37 cdSNPs are shown in solid colors (blue, red, and gray). Remaining SNPs are shown as partially translucent (blue, red, and gray). (B) The cdSNP profile of each sequence shown in (a), along with that of Clade 19B-1. Nucleotides that match EPI_ISL_418869 are denoted with a dot. (C) Maximum likelihood phylogenetic tree of representative genomes from Clade 19B-1. The focal sequence EPI_ISL_418869 is shown with an orange star. The tree was inferred under the General Time Reversible model with invariant sites with 100 bootstraps and rooted using other clade 19 genomes. Filled nodes have ≥50 per cent bootstrap support, while unfilled nodes have <50 per cent bootstrap support.

Viral recombinants are often identified using algorithms implemented in programs such as RDP4 (Recombination Detection Program version 4) (Martin et al., 2015) and RAPR (Recombination Analysis PRogram) (Song et al., 2018). Some of these algorithms incorporate all SNPs equally, regardless of how phylogenetically informative they are, and thus might be prone to falsely identifying recombinants and their predicted parent sequences when applied to sequence data where substantial clade structure is already present. For example, a preliminary analysis to detect recombinant SARS-CoV-2 genomes with RAPR identified the genome EPI_ISL_418869 as a recombination product of parent sequences EPI_ISL_422974 and EPI_ISL_422983, with an estimated significance of *P* = 0.0002 (Fig. 2A) (Korber et al., 2020a). However, seven cdSNPs are present in this genome that corresponds to neither of the two parent sequences’ cdSNPs (Fig. 2B), demonstrating that recombination between the two parent sequences is exceedingly unlikely to have generated sequence EPI_ISL_418869. Further, the cdSNP profile of EPI_ISL_418869 is a perfect match to Clade 19B-1, indicating that it is a typical sequence of that clade (Fig. 2B) and a full-genome phylogenetic analysis places this sequence firmly in Clade 19B-1 (Fig. 2C).

### 2.3 Clade-defining SNP profiles identify a preliminary set of 1,175 SARS-CoV-2 sequences that may have originated through recombination

**Figure 3.**
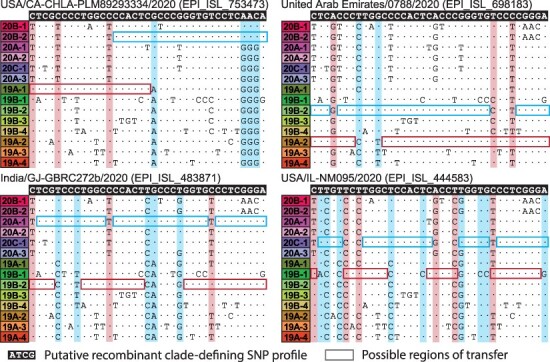
Examples of putative recombinant genomes. Possible parent clades of putative recombinants are identified by screening pairwise combinations of clades for cdSNPs that support recombination without any conflicting nucleotides. The cdSNP profile of each putative recombinant sequence (white text with black background) is compared with the profiles of all 14 clades. Nucleotides that match the putative recombinant are denoted with a dot. Regions boxed in blue and red show potential parental clades, with left and right boundaries indicating potential transfer regions. cdSNPs that support recombination are highlighted with vertical blue and red windows.

Having identified the SNPs that shape the clade structure of SARS-CoV-2, we next aimed to determine if any of the genomes available on GISAID have combinations of cdSNPs that could potentially be more parsimoniously explained by recombination. In total, we screened 537,360 full genomes and identified 1,175 potentially recombinant samples that have two or more cdSNPs supporting recombination (Supplementary Table S1). The cdSNP profiles of these genomes can be explained as the combination of two clades’ cdSNP profiles with different numbers of recombination breakpoints (Fig. 3). We chose not to consider samples with only one cdSNP supporting recombination, since the overwhelming majority of these samples are likely to be more parsimoniously explained by *de novo* mutation. To estimate the number of unique evolutionary origins of the 1175 potential recombinants we identified, we clustered these genomes into ‘genotype clusters’, which we operationally defined as sets of genomes that are at most three SNPs from each other genome-wide. Through this clustering, we found that the 1,175 potential recombinant genomes corresponded to approximately 700 unique evolutionary origins.

### 2.4 Screening of potential recombinants for evidence of clonal evolutionary histories

**Figure 4.**
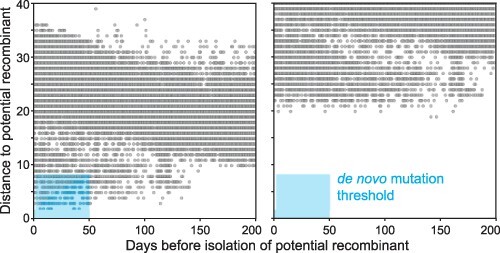
Some potential recombinant genomes may have evolved through *de novo* mutation, not recombination. All pairwise nucleotide distances between each potential recombinant genome and all non-recombinant genomes were calculated and used to identify sequences that may have evolved through *de novo* mutation (example at Left) or recombination (example at Right). The *de novo* mutation threshold is described in the text.

While some of the 1,175 genomes we identified as being potentially recombinant are likely to be true recombinants, some may have originated through *de novo* mutation at cdSNP sites. To identify this latter subset of sequences, we first calculated the pairwise nucleotide distance between each potential recom binant and all other full-genome SARS-CoV-2 sequences that were not flagged as being potentially recombinant with a collection date prior to that of the focal potential recombinant. We classified potential recombinants as *de novo* if any genomes isolated within 50 days prior to the potential recombinant could reasonably be considered a clonal ancestor. With a generation interval of 4–5 days (Ganyani et al., 2020), the 50-day window corresponds to 10–12 transmissions along a lineage resulting in the focal sequence. Assuming a substitution rate of 8 × 10^−4^ substitutions per site per year (Nextstrain: Genomic Epidemiology of Novel Coronavirus 2021), corresponding to a substitution rate of *s *= 0.066 substitutions per genome per day, a clonal evolutionary ancestor would be expected to be a distance of *st* nucleotides away from the focal sequence if collected *t* days prior to the focal sequence. Extending this logic, we computed the maximum number of nucleotide differences that would be expected between the focal sequence and an evolutionary ancestor in >99 per cent of cases. This 50 day/99 per cent probability window was then used to classify each potential recombinant as either a possible *de novo* mutant or a putative recombinant using a highly conservative threshold: if one or more genomes were observed within the window, we classified the focal sequence as *de novo* (Supplemental Table; Fig. S1; Fig. 4). In addition, if any sequence in a given 3-SNP genotype cluster was classified as *de novo*, we classified all sequences in the genotype cluster as *de novo*. Together, these conservative criteria likely result in a substantial number of false negatives, where true recombinants are instead misclassified as *de novo*.

Importantly, this is a conservative threshold since this filtering method does not consider the location of nucleotide differences. In particular, the cdSNP sites display strong conservation within each of the 14 clades and are not known to be homoplastic, so multiple mutations within the 37 cdSNP sites would be unlikely to occur through *de novo* mutation. Indeed, if mutations are distributed randomly throughout the genome, the probability of two mutations falling in any of the 37 cdSNP sites only begins to approach 1 per cent when more than 120 mutations are introduced. Consequently, this filtering method disproportionately excludes putative recombinants from early 2020 when there were fewer genome-wide nucleotide differences between circulating strains. For instance, sequence USA/CA-CZB-1437/2020 (EPI_ISL_468407) isolated on 26 March 2020 has strong support for being a true recombinant, but is excluded for these reasons.

Ultimately, we find that 358 out of the 1,175 potentially recombinant genomes are not classified as *de novo*. These 358 genomes comprise 262 genotype clusters and therefore a relatively large number of independent evolutionary events. We use these 358 genomes in all subsequent analyses, referring them as ‘putative recombinants’. Fig. 3 highlights four representative examples of these putative recombinants and their genomic regions of transfer.

Among the putative recombinants, the number of genomes identified exceeded the number of genotype clusters, indicating evidence of onward transmission. More specifically, we find that 28 of the 262 genotype clusters consisted of more than a single putative recombinant genome. While only seven genotype clusters consisted of more than three putative recombinant genomes, one cluster contained 44 putative recombinant genomes. This genotype cluster appears to have originated in the midwestern USA in October 2020, based on the geographic location of its earliest sequenced representative: EPI_ISL_848334. It then appeared to have spread to seven other US states and in a single instance to Hovedstaden, Denmark.

The putative recombinant genomes were collected from a total of 37 different countries, with the highest representation among sequences isolated from the USA and UK. Some of these genotypes contain mutations of concern, including 217 that harbor the Spike D614G substitution, 5 with N501Y, and 4 with E484K. Notably, one of the N501Y putative recombinant genotypes appears to have spread, since it was detected in three different cities in Belgium after being initially isolated in Bruges in February 2021 (EPI_ISL_961191). It is important to note, however, that we have no reason to believe that any of the putative recombinant genomes we have identified have altered transmissibility or virulence relative to their parent lineages with the same variants of concern.

**Figure 5.**
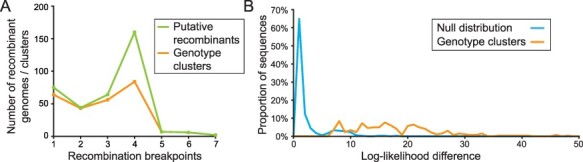
Recombination breakpoint distribution among putative recombinants. (A) Histogram showing the distribution for the number of recombination breakpoints needed to explain the cdSNP profile of a recombinant, for the 358 putatively recombinant genomes (or the 262 genotype clusters) identified. (B) Log-likelihood difference of mapping the two genome subsets of an identified recombinant and its full genome. Log-likelihood differences are shown separately for those recombinants. The null expectation, generated from a set of non-recombinant genomes, is also shown (blue).

The minimum number of breakpoints necessary to generate the putative recombinant genomes we identified ranges between 1 and 7, but 343 out of the 358 genotypes (corresponding to 94 per cent of the 262 genotype clusters) could be explained by between 1 and 4 breakpoints (Fig. 5A). This number of observed breakpoints is consistent with results from experimental coronavirus co-infections (Keck et al., 1988).

### 2.5 Evaluating the possibility of sample contamination or sequencing error

It is possible that the chimeric sequences we identified are artifacts of cross-sample contamination, co-infection, or issues related to sample processing and library preparation. Ideally, we would examine the raw reads of each recombinant to determine the likelihood of these alternatives to true recombination. Unfortunately, the raw sequencing reads are not available for the vast majority of genomes deposited on GISAID. Of the 358 putative recombinants we identified, only 25 could be linked to accession numbers on the National Center for Biotechnology Information Sequence Read Archive (NCBI SRA). Twenty-three of these genomes (92 per cent) have high-quality reads with no minor alleles detectable at any of the cdSNP sites, supporting that these genomes are recombinant. Only one genome with high coverage (≥100 read depth) is polymorphic (≥10 per cent frequency of minor variant) at any of the 37 cdSNP sites. This singular genome is consistent with either co-infection or contamination. However, eight of the genomes with no high-coverage polymorphic sites (36 per cent) have low-coverage (≤100 read depth) polymorphic sites at least one cdSNP site, indicating low sequencing coverage could have impacted the quality of the final assemblies. However, all but two of these genomes with low-coverage sites have the same cdSNP profile as high-quality/coverage genomes, suggesting many of these genomes could still be true recombinants. Overall, among those putative recombinant genomes for which raw sequencing data are publicly available, the majority are well-supported and unlikely to be due to co-infection or sequencing artifacts.

### 2.6 Positive evidence of recombination

We next sought out additional lines of evidence for recombination as the underlying process giving rise to the putative recombinant genomes we identified. Instances of recombination are most strongly supported by significant phylogenetic incongruence, with genome subsets derived from one parent falling in a different part of the phylogeny than genome subsets derived from the other parent. To assess whether our lightweight screening approach delivers putative recombinants that show significant phylogenetic incongruence between their parentally derived genome subsets, we conducted a phylogenetic placement analysis. This analysis determined whether the likelihood associated with the phylogenetic placement of the two genome subsets significantly exceeded the likelihood associated with the phylogenetic placement of the full genome. To perform this statistical assessment, we first divided each recombinant genome into genome subsets that correspond to the predicted parent clades. To do this, we identified the approximate locations of recombination breakpoints as the midpoint between cdSNPs that support recombination and then used these locations to subdivide the alignment to create two complementary genome subset sequences. These genome subset sequences each contained all genomic regions derived from one but not the other of the two predicted parent clades. We then mapped the full length and two genome subset sequences to a maximum likelihood reference tree using pplacer (Matsen, Kodner, and Armbrust 2010) and measured the extent to which subdividing the genome increases the overall likelihood of mapping (Fig. 5B). In the case of recombinants, the overall likelihood associated with mapping of the genome subset sequences will precipitously exceed the likelihood associated with mapping of the full-length sequence. Mapping 357 out of the 358 putative recombinant genomes as the two genome subsets resulted in significantly higher log-likelihoods than mapping the full genome (*P* < 0.05). To confirm that these log-likelihood differences statistically support the identification of a recombinant, we further generated a log-likelihood difference null expectation from non-recombinant genomes. To generate this null expectation, we sampled 262 non-recombinant genomes and cut them according to the same pattern of breakpoints as the 262 putative recombinant genotype clusters. Using pplacer (Matsen, Kodner, and Armbrust 2010), we then calculated the log-likelihoods of mapping the two genome subsets versus the full genome and plotted their difference as the null expectation (Fig. 5B). As expected, the log-likelihood differences from the non-recombinants were considerably smaller than those of the recombinants, indicating that subdividing genomes according to recombinant breakpoints substantially improves mapping among putatively recombinant genomes, but not among non-recombinant genomes (Fig. 5B). Interestingly, we find weak bimodality in the null expectation, which could reflect low levels of within-clade recombination not detectible by our cdSNP-based method.

**Figure 6.**
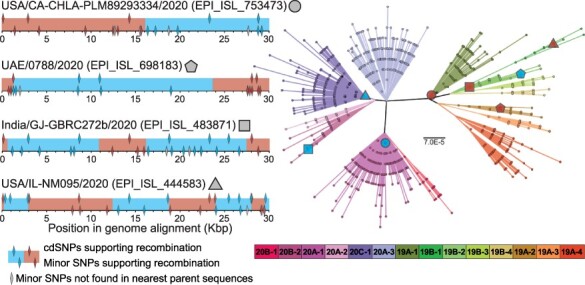
Phylogenetic placement analysis of recombinant genome regions supports parental clade prediction. (Left) The putative recombinant genomes differ in the number of recombination breakpoints necessary to explain their cdSNP profiles. The locations of cdSNPs mapping to each parent clade are shown with blue or red diamonds (top of bar). Minor SNPs from the nearest parent sequences that support or conflict with recombination are also shown (bottom of bar). Possible parent sequences from top to bottom are EPI_ISL_753557 and EPI_ISL_837758, EPI_ISL_698192 and EPI_ISL_698929, EPI_ISL_483865 and EPI_ISL_483872, and EPI_ISL_632020 and EPI_ISL_571523. cdSNPs and minor SNPs that are shared by both predicted parent clades are not shown. (Right) Genome regions highlighted in red and blue were mapped to a maximum likelihood reference tree (inferred under the General Time Reversible model with invariant sites) using pplacer (Matsen, Kodner, and Armbrust 2010). Each genome subset mapped onto the reference tree is shown with red and blue filled shapes. Minor SNPs were used to aide in identifying recombination breakpoint locations used for phylogenetic placement of these four genomes.

We further examined in detail the four putatively recombinant genomes shown in Fig. 3. Consistent with the results of the phylogenetic placement analysis, we find that for these genomes, genetic variation outside of cdSNP sites further supports recombination and additionally helps refine breakpoint boundaries. While we did not examine genetic variation outside of cdSNP sites for the other putative recombinant genomes, we did find that for all pplacer analyses, including for the sequences shown in Fig. 6, the subdivided recombinant genomes clustered closely with genomes from the predicted parent clades.

Finally, we note that recombination detection programs, such as RDP4 (Martin et al., 2015), can identify our putative recombinants as being of recombinant origin when a large number of cdSNP and minor SNPs support recombination, as shown with one example in Fig. S2. Application of this independent algorithm corroborates the cdSNP method as an initial, lightweight approach for screening a large number of genomes for possible recombinant origin.

### 2.7 Plausibility of recombinants based on geographic considerations

**Figure 7.**
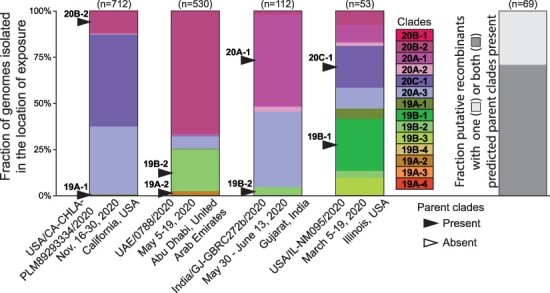
Predicted parental clades of recombinant viruses are frequently detected circulating in geographic locations of exposure prior to the collection dates of recombinants. Predicted parent clades for each listed recombinant genome are shown with arrows. The total number of genomes sequenced from each location during the specified time period is shown above each bar in parentheses.

We next sought to assess the geographic plausibility of transfer between the predicted parental clades to generate the putative recombinant genotypes identified. We therefore evaluated which clades were prevalent at the location where the putative recombinant was collected, in the 2 weeks prior to its collection (Fig. 7). For each of the 262 putatively recombinant genotype clusters, we identified the earliest sequence with complete metadata. Sixty-nine of these sequences (26 per cent) were first isolated from individuals who were exposed to SARS-CoV-2 in a country where at least 200 genomes had been sequenced in the 2 weeks prior to isolation. Of these 69 putative recombinant genotypes, both of the predicted parent clades were detected in the 2 weeks prior to isolation for 49 genotypes (71 per cent), demonstrating that co-infection with the two predicted parent clades was feasible for the majority of putative recombinants.

### 2.8 No evidence for hotspots of recombination in the SARS-CoV-2 genome

**Figure 8.**
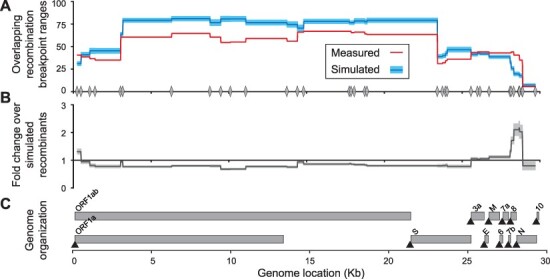
Identification of hotspots for recombination breakpoints. (A) The number of overlapping recombination breakpoint ranges at each site in the SARS-CoV-2 genome from the earliest sequences of the 262 putatively recombinant genotype clusters (red) and an equal number of simulated recombinant genomes (blue). 95 per cent confidence intervals are shown in shaded blue. The locations of the 37 cdSNPs are shown along the X-axis with gray diamonds. (B) Fold change in the number of overlapping breakpoint ranges of the observed recombinants over simulated recombinants. (C) Location of the open reading frames and transcription-regulatory sequences (black triangles) in the SARS-CoV-2 genome.

We next sought to use our set of putative recombinant genomes to ask whether recombination hotspots in the SARS-CoV-2 genome may be apparent. The limited genome diversity restricts our ability to identify discrete regions where recombination breakpoints occur. Instead, we identified ranges where breakpoints could have occurred based on the location of cdSNPs (Fig. 8A). Next, we generated a simulated dataset that was designed to represent the null expectation that recombination breakpoints occur randomly across the genome. Simulated recombinant genomes were generated such that they have the same distribution of breakpoints per genome as the GISAID recombinants, but the locations of those breakpoints in the genome were random. Each simulated genome was generated by taking two random parent clades, picking random locations in the genome to create breakpoints, and then assigning the identity of cdSNPs according to the location of those breakpoints and the parent clades used. Importantly, the probability of selecting each parent clade was proportional to its worldwide abundance in GISAID and the resulting simulated genomes were then screened using the same scripts used to screen GISAID sequences to ensure they passed the same criteria. Comparing the observed and simulated recombination breakpoint ranges indicated only a modest enrichment in the 28–29 kb region of the genome (Fig. 8B). While this region of the genome has a dense concentration of transcription-regulatory sequences (Fig. 8C), the extent of enrichment observed is not strongly supportive of these sequences promoting recombination. Thus, our results suggest that breakpoints occur randomly and are not substantially structured by genome composition or biological processes. Alternatively, our currently limited dataset of putative recombinants may simply not have the power to detect low levels of recombination hotspot activity.

### 2.9 At most 0.2–2.5 per cent of circulating viruses are recombinants

**Figure 9.**
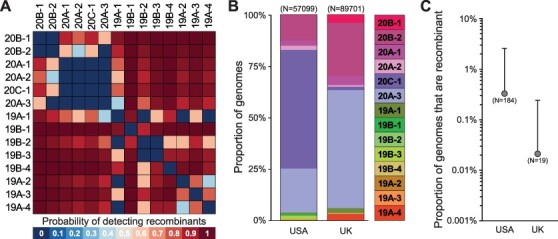
Estimation of the maximum proportion of the circulating virus population that is recombinant. (A) The probability of detecting a recombinant from pairwise combinations of parent clades when at least two cdSNPs are required to support recombination. (B) The relative abundance of each clade in GISAID from the USA and UK, calculated from SARS-CoV-2 sequences collected prior to 16 February 2021. (C) Proportion of genomes that are recombinant in the USA and UK. The top of the error bar shows the maximum estimated proportion of recombinants after accounting for local clade frequencies. The total numbers of recombinant genotypes used in each analysis are shown in parentheses.

Through our analysis we identified 1,175 putatively recombinant genomes out of >500,000 queried sequences, of which 358 were unlikely to have evolved via *de novo* mutation. This corresponds to a frequency of recombinants of 0.07 per cent. While some of these are likely to arise from sample contamination or sequencing error, some of the putative recombinants we dismissed as originating from *de novo* mutation likely instead originated from recombination. Beyond these false-positive and false-negative possibilities, it is important to note that this 0.07 per cent detected that the frequency of putative recombinants is an extreme lower bound on the frequency of recombinants in circulation for the following reason: many true recombinants will go undetected by our cdSNP detection approach because they involve two parent clades that have highly similar cdSNP profiles, thereby generating a recombinant that may have either zero or only one cdSNP supporting recombination. Indeed, the probability of detecting a recombinant depends on the number of cdSNPs that differentiate its parent clades (Fig. 9A). As such, the relative abundance of the 14 clades in a given geographic location (along with sampling intensity) determines the chance that a recombinant genome is detected if it is in circulation. By considering these factors impacting recombination detection, we were able to estimate a ceiling on the proportion of circulating viruses that are recombinant. We estimated this ceiling for the USA and for the UK separately, due to markedly different relative abundances of the 14 clades in these two regions (Fig. 9B). We chose these geographic locations due to their large sequencing efforts relative to other locations. For these two countries, we estimate at most 0.2–2.5 per cent of circulating viruses were recombinant (Fig. 9C).

### 2.10 Continued surveillance

Since we downloaded the 537,360 complete SARS-CoV-2 genomes available on GISAID for the analysis in this paper on 16 February 2021, additional 1.3 million genomes have been deposited as of 1 June 2021. As this number continues to grow, continued surveillance will greatly benefit from a fast and lightweight method for screening new genomes for recombinant sequences. Most existing recombination detection tools are not well suited to the combination of large database sizes and low levels of genome-wide diversity, both of which characterize current SARS-CoV-2 sequence datasets. Although our approach was successful in screening over half a million genomes, its efficiency and user accessibility are limited by the large resource demands needed to generate whole-genome alignments. With this need in mind, we developed a lightweight version of our pipeline that requires only BLASTn (Altschul et al., 1990) and python to generate local alignments to identify cdSNPs. This method, termed cladeSNP-blast, can screen 10 kb genomes in 15 minutes on a 3.5-GHz single-core CPU with no loss in specificity. The code is available on GitHub (https://github.com/davevanins/Sars-CoV-2_CladeSNP). Potential recombinants identified with this pipeline, however, should undergo additional screening for clonal evolutionary histories, as we have done in our above analyses.

## 3. Discussion

The small number of polymorphic sites in the SARS-CoV-2 genome that are phylogenetically informative means detecting recombinant genomes is difficult and highly dependent on the identity of the parent clades. By identifying the nucleotide changes that underpin the clonal phylogeny of SARS-CoV-2, we established criteria for identifying potential recombinant genomes. We then outlined a stringent secondary screen to remove potential recombinants that could feasibly have arisen by *de novo* mutation. We analyzed 537,360 SARS-CoV-2 genomes from GISAID and found 358 viral sequences that contain evidence of recombination. These genomes have rearrangements of multiple cdSNPs that support recombination between clades, lack potential clonal ancestors based on pairwise nucleotide distance, and are typically isolated in countries where the predicted parent clades are prevalent. Although the number of recombinants depends heavily on the criteria used to distinguish true recombinants from *de novo* mutation or other sources of error, we estimate that the number of circulating recombinant viruses remains low (<0.2–2.5 per cent).

While we were able to identify 358 putatively recombinant SARS-CoV-2 genomes, they represent an extremely small fraction of the genomes available on GISAID (0.07 per cent). Although our *de novo* mutation filtering is likely removing some true recombinant sequences, particularly those isolated early in the pandemic when genome-wide diversity was low, the data strongly suggest that recombinants remain rare but are circulating at low levels. This observation supports reports that have found no evidence of widespread recombination among SARS-CoV-2 genomes (Maio et al., 2020; Nie et al., 2020; Wang et al., 2020). Indeed, examining the pattern of cdSNPs suggests none of the 14 clades we identified emerged through recombination (Fig. 1). The only site that does not strictly follow the pattern of vertical descent is C14805T, which occurs in both Clades 19A-4 and 19B-4. However, none of the seven other cdSNPs that differentiate these clades support recombination, suggesting C14805T is a homoplastic site.

The low frequency of recombinant sequences in GISAID could be due to multiple factors. First, due to the limited genetic diversity of SARS-CoV-2 at this point in time, a large fraction of viral recombinants may not be detectable. This is particularly the case if the parental clades share a large number of cdSNPs (Fig. 9A). However, this cannot be the sole factor, given that our ceiling estimates of 0.2–2.5 per cent take into consideration the inability to detect recombinants rising from pairs of parent clades. Second, recombinant genomes may be rare because co-infections occur only rarely. Co-infection may be infrequent for SARS-CoV-2 given the acute nature of the infection and that some geographic regions (but not others) managed to keep the level of virus circulation low during this first year of the SARS-CoV-2 pandemic. Third, recombinant genomes could be rare because they are transmitted infrequently. For instance, when co-infections do occur, recombinant genomes may evolve late in the infection, resulting in rare onward transmission.

It is likely that a portion of the putative recombinants we identify here are non-recombinant sequences that reflect issues with library preparation, sequence depth limitations, and co-infection or contamination. For instance, seven genomes include breakpoints within the triple nucleotide GGG to AAC substitution at Sites 28,881–3, and although this is technically possible, it is potentially an indication that these genomes have poor sequence quality. Our analysis nevertheless suggests the majority of the putative recombinants we identified are based on high-quality sequences with multiple independent lines of evidence supporting the feasibility of recombination. In particular, 28 of the 358 putative recombinant genotypes occurred more than once in GISAID, and the majority of these genotypes were sequenced multiple times by different laboratories in the same country or independently isolated in other countries. These observations suggest that many of the sequences we identified cannot be accounted for by sequencing artifacts. This is further supported by our analysis of raw sequencing data where available. However, since raw sequence data are not available for the vast majority of putatively recombinant sequences considered here, our conclusions are based on the assumption that the majority of these genomes are free from contamination and technical errors. Although the number of recombinant genomes in GISAID is difficult to define precisely, small variations in the total number do not substantially affect our estimates of the maximum proportion of recombinant viruses that are currently circulating. Further, our ceiling estimates for circulating recombinants do not increase substantially when we use a less stringent *de novo* mutation threshold (results not shown). Without excluding any possibly *de novo* mutants, the maximum proportion of circulating viruses that are recombinant increases only from 0.2–2.5 per cent to 1–5 per cent.

Since one of the limiting factors in identifying recombinant genotypes is the small number of phylogenetically informative sites, it is tempting to assume that it will become a progressively easier process as mutations continue to accumulate. However, the ability to detect recombinants depends on the identities of the circulating clades and these may change over time, in part due to viral adaptation. For example, the probability of detecting a recombinant is highest between Clade 19 and 20 parents and lowest between Clade 20 parents (Fig. 9). However, Clade 19 viruses have become progressively rarer while the D614G harboring Clade 20 viruses have disproportionately driven waves of infection around the world. As a result, recombinants have become more difficult to detect than when Clade 19 and 20 viruses co-circulated more uniformly. Similarly, recent waves of infection driven by the N501Y.V1 lineage has reduced the diversity of circulating viruses and further limited our ability to detect recombinant genomes. Accordingly, we expect that the identification of SARS-CoV-2 recombinant genomes may continue to be difficult as novel adaptive mutations continue to drive new waves of infection.

Ultimately, our results suggest that recombination between SARS-CoV-2 strains is occurring, but these chimeric genotypes remain rare. Although we identify a small number of recombinant genotypes that are actively circulating, we have no reason to expect that these lineages—or any other recombinants identified here—have increased transmissibility or virulence. Yet, as novel mutations that influence transmissibility or threaten to limit the efficacy of vaccines continue to evolve and spread, the potential for recombination to facilitate merging these mutations into a single background will continue to increase. Given our finding that recombination is already occurring in SARS-CoV-2, surveillance efforts and real-time analyses to detect recombinants, such as the one here, should be sustained to monitor the circulation and potential spread of high-fitness recombinant genotypes.

## 4. Materials and Methods

### 4.1 Genome quality filtering and alignment

Genomes were downloaded from the GISAID genome database (Elbe and Buckland-Merrett 2017) and filtered to exclude low-quality sequences. All genomes were trimmed relative to Positions 118 and 29,740 in the NCBI reference sequence (accession NC_045512) because these regions are inconsistently assembled between genomes and increase resource demands and uncertainty in the following steps: to trim genomes at these locations precisely prior to whole-genome alignment, the 118 and 29,740 sites were identified using BLASTn (Altschul et al., 1990). After trimming, genomes with less than 1 per cent Ns and a final length greater than 29,610 bp and less than 29,660 bp were included in further analysis. Genomes were aligned to the NCBI reference sequence genome using MAFFT v7.464 (Katoh and Standley 2013), using the option ‘keeplength’ to exclude any insertions not present in the reference sequence. Excluding insertions reduced resource limitations and enabled parallelization since all genomes are aligned to the same reference.

### 4.2 Identifying clade-defining SNPs in SARS-CoV-2 genomes

Clades were identified as monophyletic groups with at least 50 per cent bootstrap support within a maximum likelihood phylogenetic tree built from 6,536 unique high-quality genome sequences using PhyMLv3.1 (Guindon et al., 2010). Reference genomes were picked by clustering all genomes available on GISAID from February 1 to 1 July 2020, based on their pairwise distances on a neighbor-joining tree constructed from a whole-genome alignment. Representative strains were picked to minimize redundancy while maximizing the total sampled diversity. Accession numbers of these reference genomes are available on our GitHub repository (https://github.com/davevanins/Sars-CoV-2_CladeSNP). Clades were named according to the Nextstrain clade to which they belong, with added suffixes of ‘-1’, ‘-2’, etc. to denote clades at finer resolution than those available under the Nextstrain system. cdSNPs were defined as sites that are practically monomorphic within each clade (with >95 per cent of the clade members carrying the same allele) but are polymorphic across clades, such that the dominant allele in a given clade is not dominant in all clades. For example, 99.5 per cent of the members of Clades 20B-1 have a thymine (T) at cdSNP site 23,731 while the remaining 13 clades each have a cytosine (C) at that same site in over 95 per cent of their clade members (Fig. 1C). Recombinant genomes were identified by comparing the cdSNP profiles of each query sequence against the profiles of the 14 clades. Any sequences with at least two cdSNP differences from the nearest clade cdSNP profile were screened to determine that the genotype could be explained by recombination between two parent clades. This minimum distance to the nearest clade cdSNP profile represents the minimum number of cdSNPs supporting recombination. All genomes with two possible parent clades based on cdSNPs that pass all other quality thresholds were considered potentially recombinant. The number of breakpoints in a putative recombinant was estimated as the minimum number of breakpoints required to explain the parental origin of the genome’s cdSNPs. In total, 537,360 genomes were screened. The list of their GISAID accession numbers can be found on the GitHub repository for the analyses contained within this manuscript (https://github.com/davevanins/Sars-CoV-2_CladeSNP).

### 4.3 Screening potential recombinant genomes for *de novo* mutants

Potential *de novo* mutants were identified by determining if any non-recombinant sequence fell within a nucleotide distance threshold such that it could be considered the clonal ancestor of a potential recombinant. First, the pairwise distance between each potential recombinant and all non-recombinant SARS-CoV-2 genomes was calculated. Only non-recombinant genomes with no ambiguous nucleotides were included, and sites in the potential recombinant genome that included ambiguous nucleotides were ignored while calculating the total pairwise distance. The nucleotide distance threshold was calculated as the maximum distance that would be expected in >99 per cent of cases given a substitution rate of 8 × 10^−4^ substitutions per site per year (Nextstrain: Genomic Epidemiology of Novel Coronavirus 2021), within a time frame of 50 days prior to the collection date of the potential recombinant. This distance threshold was calculated using the Poisson inverse cumulative distribution function. Any sequence within this distance/time threshold was flagged as a possible *de novo* mutant. Additionally, to account for related potential recombinant genomes, all potential recombinant genomes that differ by ≤3 SNPs genome-wide were grouped into genotype clusters. If any genome within a genotype cluster was flagged as possibly *de novo*, all genomes within that cluster were flagged as being possibly *de novo*.

### 4.4 Phylogenetic placement analysis

Phylogenetic placement was performed to provide statistical support for recombination. After predicting the parent clades that minimize the number of cdSNP differences and recombination breakpoints, putative recombinant genomes were subdivided according to the midpoints of the recombination breakpoint ranges. These genome subsets were then mapped onto the maximum likelihood reference phylogenetic tree using pplacer (Matsen, Kodner, and Armbrust 2010), which provides their log-likelihoods of placement along particular branches. Placement on the reference tree was visualized using Interactive Tree of Life (Letunic and Bork 2019). Significance of mapping was determined by the log-likelihood difference between the combined log-likelihood of mapping both genome subsets and the log-likelihood of placement for the full-length genome. Putative recombinant genomes were compared to a null distribution from non-recombinant sequences. The null distribution was generated by subdividing the genomes of 262 random non-recombinant sequences according to the breakpoints inferred for the observed recombinants, sampled without replacement.

### 4.5 Quantifying recombination breakpoint frequency

Recombination breakpoint frequency enrichment was quantified by comparing the number of overlapping breakpoint ranges in the 262 putatively recombinant genotype clusters with ranges derived from simulated recombinant genomes with random breakpoint locations. Breakpoint ranges were defined as the full region between any two cdSNPs that were predicted to come from different predicted parent clades. To simulate recombinant genomes, we picked two random parent clades and picked random locations throughout the genome to place breakpoints. Based on the locations of the random breakpoints and the 37 cdSNPs, we created cdSNP profiles for each simulated genome by first randomly picking which parent clade the very first cdSNP originated from and then assigning the remaining cdSNPs of the recombinant according to the locations of the breakpoints. The probability of picking any one clade was proportional to its abundance in GISAID. Simulated recombinants were generated such that they had the same distribution of breakpoints per genome as the observed recombinants. To do this, recombinants were generated iteratively by creating genomes with one random breakpoint until enough simulated genomes with ≥2 cdSNPs supporting recombination were identified using the same code used to screen GISAID genomes. This process was then repeated for 2–10 random breakpoints, where the number of detected breakpoints was required to match the number inserted. Simulated recombinants with redundant cdSNP profiles were discarded. Fold change in the number of overlapping breakpoint ranges in the GISAID recombinants relative to the simulated recombinants was calculated in 10 nucleotide-long bins across the genome. Ten separate simulations were performed to calculate 95 per cent confidence intervals.

### 4.6 Calculating the ceiling on the proportion of circulating virus that is recombinant

To statistically estimate the maximum proportion of recombinant viruses circulating in a population, we first calculated the probability of detecting a recombinant arising from parent clades *i* and *j*, where *i* and *j* take on values between 1 and 14, and the number of parent clades identified using a 50 per cent bootstrap support cutoff value. We define *m* as the number of cdSNP sites at which parent clades *i* and *j* differ from one another. We define *T* as the threshold number of cdSNP differences required to identify a recombinant. (In our analyses, we set *T* = 2.) If *m* < *T*, then the probability of observing a recombinant between these parent clades was set to zero. If *m* ≥ *T*, the probability of observing a recombinant between two parent clades *i* and *j* was calculated as follows:


$$\begin{equation*}{r_{ij}} = \mathop \sum \limits_{k = T}^{m - T} bino\left( {k,m, 0.5} \right)\end{equation*}$$

where *bino*(*k, m*, 0.5) yields the probability that a recombinant between parent clades *i* and *j* has exactly *k* of the *m* distinguishing SNP sites derived from one parent and the remainder derived from the other parent. This calculation assumes an infinite number of recombinant breakpoints, such that cdSNPs along the genome each have equal probability to belong to parent clade *i* as parent clade *j*. This is particularly useful for estimating the ceiling because it is the least conservative assumption. Fig. 9A shows these probabilities for the *T* = 2 cdSNP threshold used throughout our analysis.

For a given geographic region, we then calculated the frequency of each of the 14 clades from sequences deposited in GISAID prior to 16 February 2021. We denote the frequency of clade *i* as *p*_i_. These frequencies are shown in Fig. 9B for the USA and UK.

To estimate the ceiling on the proportion of recombinant genomes in circulation, we first calculated the overall probability *D* of a sampled virus being detected as a recombinant under the assumption that a proportion ${P_r}$ of circulating viruses are recombinant. This overall probability is given by:


$$\begin{equation*}D = \mathop \sum \limits_{i = 1}^{14} \mathop \sum \limits_{j = 1}^{14} {P_r}{p_i}{p_j}{r_{ij}}\end{equation*}$$

Given *N* sampled genomes from a region, we then calculated the 95 per cent confidence interval for the number of recombinant genomes that would be detected among the number of sampled genomes. This is given by the binomial inverse cumulative distribution function, evaluated at 0.025 and 0.975, with the total number of trials being given by *N* and the probability of success being given by *D*. We determine the ceiling as the value of ${P_r}$ that yields a lower bound on the 95 per cent confidence interval that exceeds the number of observed recombinants in the data, *n*.

### 4.7 Assessing assembly quality of recombinant genomes

Raw sequencing reads were downloaded from the NCBI SRA and processed using the BBTools feature ‘bbduk’ to trim contaminating adapter and PhiX sequences and remove reads shorter than 25 nucleotides long and any reads with poly-a sequences. Reads were then trimmed using trim galore with default settings. The remaining reads were mapped to the SARS-CoV-2 reference genome (accession NC_045512) with ‘bbmap’ with a minimum average quality of 25. Samtools was used to generate a pileup formatted alignment, and assembly quality at the 37 cdSNP locations was assessed by parsing that file using custom python scripts. Assemblies were assessed for the quality and depth of coverage for reads mapped to the 37 cdSNP sites. To determine if co-infection or contamination could have influenced the consensus assembly, indicated by having low-frequency alleles (>10 per cent) at any of the 37 cdSNP positions.

## Supplementary Material

veab059_Supp

## Data Availability

All custom computer code necessary to reproduce our results are available on GitHub (https://github.com/davevanins/Sars-CoV-2_CladeSNP).
