# Supplementary figures and images for: Recombinant SARS-CoV-2 genomes circulated at low levels over the first year of the pandemic
Source: Virus Evol. 2021 Jul 15;7(2):veab059. doi: 10.1093/ve/veab059 (PMC8344435; doi:10.1093/ve/veab059)

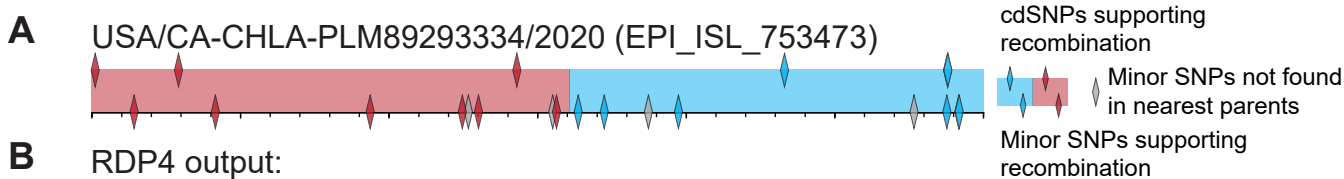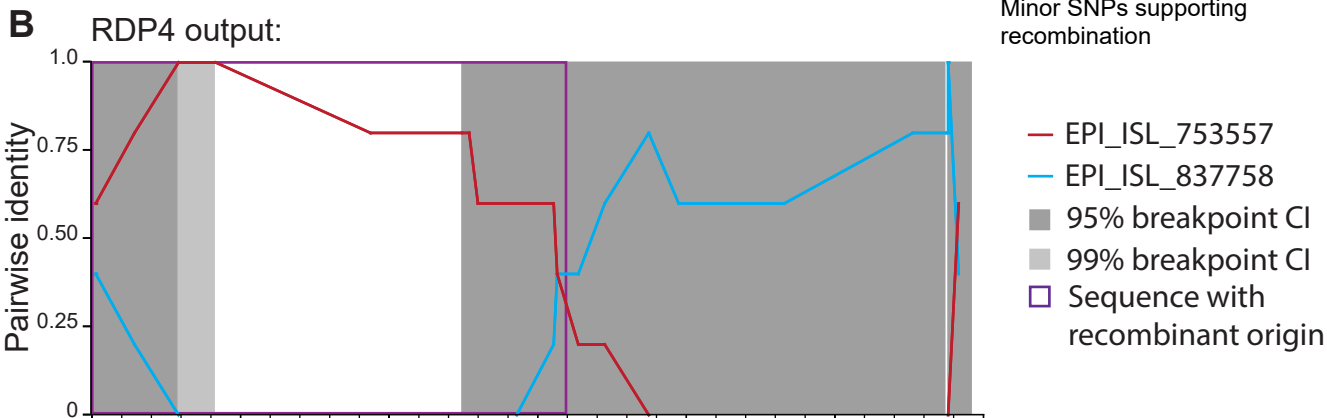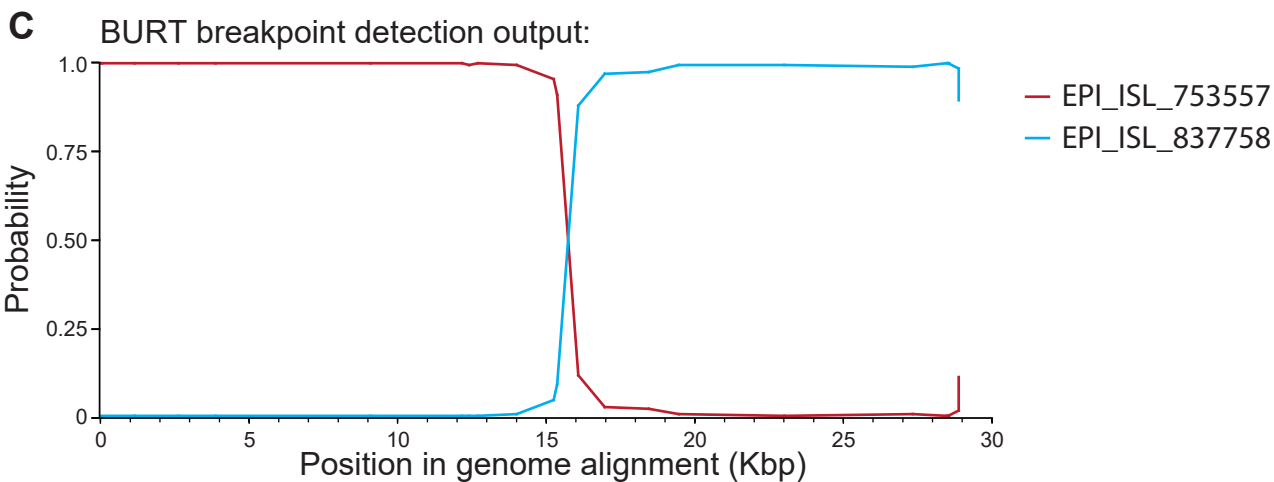

Supplement: veab059_Supp [file veab059_supp.zip › FigS2.pdf]
